# Does Energy Efficiency Realize Energy Conservation in the Iron and Steel Industry? A Perspective of Energy Rebound Effect

**DOI:** 10.3390/ijerph191811767

**Published:** 2022-09-18

**Authors:** Rongxin Wu, Boqiang Lin

**Affiliations:** School of Management, China Institute for Studies in Energy Policy, Xiamen University, Xiamen 361005, China

**Keywords:** energy efficiency, energy services, the rebound effect, energy conservation, environmental protection

## Abstract

The energy rebound effect may weaken the impact of energy efficiency improvement on energy consumption. Therefore, the rebound effect is an important consideration in energy and environmental policies. This study takes the iron and steel industry as the research object, which is a large energy consumption sector in China, and the improved technique is used to estimate the energy rebound effect. The study constructs the dynamic energy efficiency utilizing provincial data from 2000 to 2019. The energy rebound effect from factor substitution and output expansion is then calculated. The research further discusses regional differences in the energy rebound effect. The results indicate that the technical progress of the iron and steel industry promotes energy efficiency improvements. The eastern region shows the best energy efficiency performance, followed by the central area, and the western region performs the worst in energy efficiency. The industrial energy rebound effect is 0.4297, which partially offsets the energy reduction caused by energy efficiency improvements. Factor substitution and output growth produce the industrial energy rebound effect. Furthermore, the rebound effect exhibits distinct geographical features. The policy suggestions are finally proposed to mitigate the industrial rebound effect and achieve energy and carbon reductions.

## 1. Introduction

The contradiction between energy demand growth and energy resource scarcity is becoming increasingly prominent. This contradiction also intensifies energy constraints on the economy’s sustainable development. In the new century, global energy consumption increased rapidly. According to the International Energy Agency, global energy consumption in the year 2019 was 606,490 PJ (10^12^ J), representing a 44.82% increase versus the year 2000 [1]. The industrial sector is the largest terminal department of energy use, accounting for about 19.95% of the global energy use [1]. In addition, the global energy shortage problem is becoming more severe with the rising energy price [2]. The massive energy use also brings about increasingly severe environmental problems. Consequently, energy and environmental issues have long been an important topic of concern for the international community [3,4].

The economic development in China has become more dependent on energy and the environment in the past four decades [5,6,7]. China consumes 3.3 times more energy than India, the world’s third largest energy user [1]. China’s massive fossil energy consumption is directly responsible for the deteriorating ecological environment [8,9]. The main point is that fossil fuels makes China emit more CO_2_ than any other country, accelerating global warming [10]. What’s worse, particulate matter (PM2.5), sulfur dioxide, nitrogen oxides, and other pollutants from energy consumption generate fog and hazy weather in China. The Chinese government has pledged to construct a resource-efficient and environment-friendly society [11]. But the existing energy and environmental scenario are impeding its sustainable growth.

**Figure 1:**
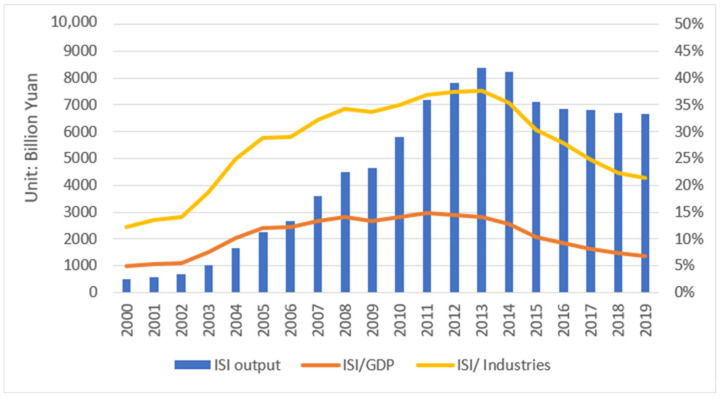
The output value of China’s ISI and its proportion in GDP and whole industries. Source: China Industry Statistical Yearbook, 2001–2020.

**Figure 2:**
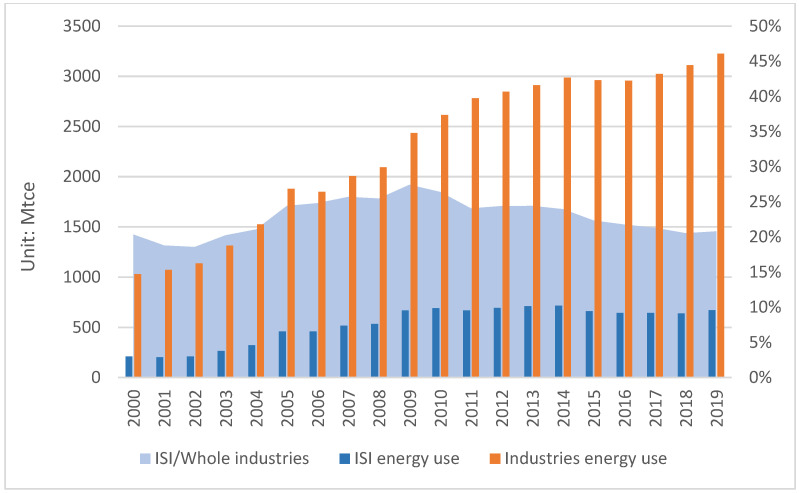
Energy use in China’s ISI and its proportion in whole industries. Source: China Statistical Yearbook, 2001–2020.

China’s iron and steel industry (ISI) forms a complete production system, and its industrial output ranks first in the world. Figure 1 presents that after entering the 21st century, the development of the ISI can be roughly divided into two stages. From 2000 to 2013, it witnessed a period of large-scale and rapid development, and the ISI has gradually transformed to scale reduction and high-quality development since 2014. The important role played by the ISI in China’s economic development cannot be ignored. The contribution rate of the ISI to the national economy was nearly 15% at its peak and above 5% in most years. The ISI shows a more obvious role in promoting the whole industrial economy, with an average annual contribution rate of more than 25%. The rapid progress of this industry is supported by energy and environmental resources. It consumes a lot of energy resources [12], which is shown in Figure 2. The energy consumption of the ISI in 2019 was 670.47 Mtce, more than three times of that in 2000. The energy used to produce iron and steel accounts for a large proportion of energy in the whole industrial sectors. In China’s industrial sectors, coal-dominated energy use usually leads to a large amount of CO_2_ emissions. China’s ISI has huge potential for energy-saving and CO_2_ reduction [13]. The Central Economic Work Conference held in 2015 kicked off the supply-side structural reform represented by steel production reduction. In April 2019, the government released the Opinions on Promoting the Implementation of Ultra-Low Emissions in the Iron and Steel Industry. It provided policy support for iron and steel firms to achieve ultra-low emissions. According to the China Iron and Steel Association, the ISI will achieve CO_2_ emissions peaking by 2025. The ISI faces stricter environmental regulations to control energy use and carbon emission.

The effectiveness of energy conservation and CO_2_ reduction contributes to green industrial development. The benefits of green production in the ISI can be summarized in two aspects. On the one hand, there is currently a risk of the oversupply of steel products in China. Green development is a key driving force for the transformation and upgrading of the ISI. The green transformation of firms can eliminate outdated capacity and promote China to become a high-quality iron and steel producer. On the other hand, green development requires reducing energy intensity and improving energy structure. It is an important step to address the environmental pollution problem caused by iron and steel production at the source. For these two reasons, the energy and environmentally friendly development of this industry attract stakeholders’ attention. In September 2019, fifteen large Chinese steel firms jointly issued the Declaration on the Green Development of Chinese Steel Enterprises. This practice shows that by taking initiatives in green production, iron and steel firms can achieve harmony with the natural society.

Improving energy efficiency is considered a cost-effective tool to achieve energy and ecological-friendly targets [14,15]. Under the 2 °C temperature control target, 40% of carbon reductions by 2050 will come from improved energy efficiency [16]. Many countries have taken action to reduce energy consumption and improve energy efficiency [17]. When it comes to energy efficiency and environmental protection, China is more actively involved. However, it is still too early for the Chinese government to link energy efficiency initiatives to the goals of energy conservation. The impact of energy efficiency on energy consumption is uncertain. Energy efficiency improvements control energy use while also stimulating energy consumption. It is mainly because the rebound effect makes the policy effects of energy efficiency uncertain. The energy saved by the energy efficiency is often partially or wholly offset by the rebound effect through substitution and output channels [18]. Therefore, economic policies and technical measures to improve energy efficiency may deviate from people’s expectations [19], and the rebound effect prompts the re-examination of the policy perspective of reducing energy consumption. Can energy efficiency achieve goals of saving energy and environmental protection? This is an issue of interest to many policymakers. The extent to which energy efficiency improvements reduce energy consumption depends mainly on the rebound effect size.

Currently, policies are often formulated focusing on the technical aspects of the potential for energy savings, ignoring the linkages between a firms’ production behaviors and the socioeconomic system. As a result, the prospect of energy conservation is often overestimated, and energy-saving and environmental-friendly policies are not working. Reliably quantifying the rebound effect is essential to estimate the actual energy savings resulting from energy efficiency policies. To present a practical plan to minimize energy usage, this research measures the energy rebound effect of the ISI from the producer’s perspective. The study aims to answer three questions concerning the ISI. (1) What is the present state of industrial energy efficiency? What are the features of energy efficiency performance and influence factors in different regions? (2) Is there an energy rebound effect in the context of energy efficiency, and what is the influence mechanism? In particular, how do factor substitution and output growth contribute to the rebound effect? (3) Does the energy rebound present heterogeneously in different regions of China, where economic and industrial development patterns range greatly? The quantitative investigation of the energy rebound effect aids in explaining energy use in the ISI.

The innovation of this article includes three layers. (1) Most earlier research estimates energy price elasticity as the rebound effect because of limited data on energy efficiency and energy services. However, if the energy price is strictly regulated, the variation range may be negligible. The use of energy price elasticity estimates may be biased. Drawing on the improved method of Liu et al. [20], this study estimates the rebound effect regarding the elasticity of energy services consumption concerning their prices. This method is derived from the concept of the rebound effect and has the potential to increase the estimation validity. (2) Existing studies mostly describe the industrial rebound effect in terms of factor substitution [21], but ignore the minor effect of the output expansion [22]. Figuring out the mechanism of the rebound effect is more important than calculating its magnitude [23]. This study estimates channels of factor substitution and output expansion. It enables us to accurately identify the impact mechanism of the rebound effect and provide timely empirical support. (3) In reality, the rebound effect has not been incorporated into designing energy conservation policies in China’s industries, and the government ignores this problem when implementing energy policies. The ISI is essential in energy saving and environmental protection. Therefore, the research on the industrial rebound effect can provide more targeted suggestions. Additionally, the findings of this study provide an important reference for energy conservation in energy-intensive industries.

The study’s following chapters are structured as follows. Section 2 revisits the classical literature on the energy rebound effect. Section 3 builds the econometrics model and presents variables and data. The results are analyzed and discussed in the next chapter; the final chapter concludes the research and gives suggestions.

## 2. Literature Review

### 2.1. Definition and Classification of the Energy Rebound Effect

The Jevons’ Paradox is the earliest study of the rebound effect, which is one of the most important topics in energy economics. In other words, improving fuel (coal) efficiency tends to increase (rather than decrease) fuel use [24]. However, it didn’t attract much notice at that time. Later, Khazzoom [25] and Brookes and Grubb [26] revisited the Jevons’ Paradox for energy use in society, and extended its concept to the macroeconomic level. They argued that energy efficiency improvement did not necessarily reduce energy demand but may increase energy services across the economy. Their findings are known as the Khazzoom–Brookes (KB) hypothesis [27]. If the real energy prices don’t change, technological advances may boost energy demand. Although some researchers questioned the KB hypothesis [28,29], Saunders [27] demonstrated its existence in the neoclassical growth model.

In the new century, the major problem of energy security prompts people to explore energy-saving means actively. Under such a background, the rebound effect received greater attention. Berkhout et al. [30] intuitively interpreted the rebound effect as a percentage of the predicted energy efficiency improvement potential. However, their definition is difficult for empirical analysis because both the expected and actual energy savings involved are difficult to estimate by economic methods. Some pieces of literature further studied its classification. Greening et al. [18] divided its impact mechanisms based on the economy’s response to technological progress. That is, the direct rebound effect, the indirect rebound effect, and the economic-wide rebound effect. Of these, the latter category covers the first two types of the energy rebound effect [31]. Saunders [32] and Saunders [33] developed the study of the rebound effect and divided it into backfire to super energy savings according to its magnitude. The direct rebound effect is the focus of their articles. Sorrell et al. [34] reviewed the direct rebound effect, including research levels, data types, model structures, functional forms, and estimation methods. But their research focused on the household sector and therefore cannot be directly applied to other sectors and the whole economy.

### 2.2. Research Methods of the Rebound Effect

The existing studies provide a rich methodological basis for exploring the rebound effect. In summary, there are three main approaches, which include the quasi-experimental method, computable general equilibrium (CGE), and the econometric model.

The quasi-experimental method measures the energy services demand changes before and after the energy efficiency improvement. However, this approach does not use control groups or control confounding variables and cannot control for other factors that may impact energy services demand [35]. Due to the difficulty of measuring the demand in the field, deviations are inevitable [35]. This strategy is employed mainly in early rebound effect investigations, with the majority of these studies focusing on house heating [36].

It is also common to apply the CGE model to the macroeconomic level in the literature [37,38]. Broberg et al. [39] used the CGE in the Swedish economy and discovered a 5% increase in industrial energy use efficiency would result in an economy-wide rebound effect of 40–70%. The substitution elasticity and cost are significantly influenced, but the energy efficiency improvements in the energy sector have little impact on the rebound effect. Zhou et al. [40] constructed a static multi-sector CGE model for China. They concluded that different energy varieties have a positive rebound effect. Kulmer and Seebauer [41] adopted a multi-sector static CGE model for the Austrian economy and added six heterogeneous household groups into the model. They found a 10% increase in household fossil energy efficiency that had a 65% economy-wide rebound effect. The indirect effects among different economic sectors play an important role. Khoshkalam and Sayadi [42] explored how energy efficiency affects Iran’s rebound effect. They concluded that different energy efficiency shocks (5%, 7%, and 10%) had positive rebound effects on Iran’s sectors. In the short run, the substitution channel is the most influential component. The CGE model has the advantage of taking into account the influencing aspects of energy efficiency to guarantee a more robust outcome. However, the downside is that the social accounting matrix must be calculated and balanced [43]. The model’s output is sensitive to parameter settings so it will inevitably encounter the problem of having too many parameters.

More researchers develop econometric models nowadays to carry out their research. Econometric approaches allow for a more precise estimate with the use of production functions. These techniques can also be altered in accordance with certain theoretical presumptions. In addition, these methods can be modified according to different theoretical assumptions. It is important to note that the setting of the specific production function form may affect the results [32]. Yang and Li [44] used time series data to study China’s power generation sector with dynamic OLS and seemingly unrelated regression. Their estimated rebound effect for the power generation sector is 16%. Liu et al. [45] applied the economic accounting method and concluded that the rebound effect of the transportation department was 68.3% during 1981–2015. Using the dynamic panel quantile regression, Zhang et al. [46] found that the rebound effects of China’s road passenger transport are almost equal from time’s perspective, and the energy-saving effect in western China is the best.

### 2.3. Empirical Experience from the Production Side

The rebound effect is widely acknowledged in the academic world, although its significance is frequently debated. The academic discussion over the rebound effect focuses mostly on its causes and magnitude. The literature thoroughly investigates its effect magnitude and mechanism from many angles. Scholars of the neoclassical growth theory explain the formation mechanism of the rebound effect from the viewpoint of consumer and producer theory. Some studies from the consumer side focus on household sectors, such as in [47,48]. But more scholars investigate the rebound effect of industrial sectors. Their research from the perspective of producers is enlightening. Li et al. [49] concluded that the rebound effect was 88.42% in China’s industries and that investment-driven economic growth was detrimental to energy conservation. Craglia and Cullen [50] used a more detailed dataset of UK vehicle airworthiness tests between 2006 and 2017 to study the rebound effect. They further investigated the differences in rebound effects by geographic location and vehicle type. Sorrell and Stapleton [51] reviewed the long-term rebound effect of UK road freight. They estimated the energy rebound effect to be 61%, which is almost twice the estimated result by [52]. Du et al. [53] found that the rebound effects of different transport modes in China vary widely, with the aviation sector much greater than the road sector. They also concluded that tax and energy structure adjustment could reduce the rebound effect.

For China’s ISI, the existing research focuses on energy consumption and energy efficiency (such as in [54,55,56]). However, there are few works of literature to evaluate the energy conservation effect of ISI from the perspective of the rebound effect [21]. The energy rebound effect affects energy consumption by two channels [18]. The economic development and industrial distribution vary differently in China, so it is necessary to study the rebound effect in this context. Based on the literature review and discussion, we propose three hypotheses for the ISI.

**H1.**  *The energy rebound effect exists in the ISI, and energy efficiency improvement can not fully achieve energy conservation goals.*

**H2.**  *The impact of factor substitution on the rebound effect is greater than that of output expansion.*

**H3.**  *The rebound effect shows up differently in different regions.*

This study draws on the idea of a two-stage estimation approach [57]. The first stage estimates the industrial energy efficiency and effective energy service, and the second stage calculates the rebound effect from two channels in different regions.

## 3. Model Construction and Data

### 3.1. Measuring Energy Efficiency

In the first stage, we construct a dynamic energy efficiency indicator, namely the Malmquist energy performance index. Caves et al. [58] first defined the Malmquist productivity index to reflect the firms’ productivity changes before and after the time. Färe et al. [59] further constructed the Malmquist index to measure productivity change. Referring to the study of [60,61,62], we assume that N decision-making units in the ISI. The production technology set in period τ is described as.

(1) $$P_{\tau }=\left\{\left(K,L,E,Y\right):\left(K,L,E\right)\;\mathrm{can}\;\mathrm{produce}\;Y\right\}$$

where the industry uses capital (K), labor (L), and energy (E) to produce the desired output (Y).

Using the DEA model, the production technology can be written as:

(2) $$\begin{array}{l} P^{\tau }=\{\left(K,L,E,Y\right):\sum_{t=1}^{\tau }\sum_{n=1}^{N}\lambda_{n}^{t}K_{n}^{t}\leq K\\ \;\sum_{t=1}^{\tau }\sum_{n=1}^{N}\lambda_{n}^{t}L_{n}^{t}\leq L\\ \;\sum_{t=1}^{\tau }\sum_{n=1}^{N}\lambda_{n}^{t}E_{n}^{t}\leq E\\ \;\sum_{t=1}^{\tau }\sum_{n=1}^{N}\lambda_{n}^{t}Y_{n}^{t}\geq Y\\ \;\lambda_{n}^{t}\geq 0,N=1,\cdots ,N,t=1,\cdots ,\tau \} \end{array}$$

This study requires that the energy input be reduced as much as possible within the production possibility set, while keeping other inputs and outputs constant. Following the study of [63], this study constructs the Malmquist energy performance index (MEPI).

(3) MEPIiτ,τ+1=[DEτ(Kiτ,Liτ,Eiτ,Yiτ)×DEτ+1(Kiτ,Liτ,Eiτ,Yiτ)DEτ(Kiτ+1,Liτ+1,Eiτ+1,Yiτ+1)×DEτ+1(Kiτ+1,Liτ+1,Eiτ+1,Yiτ+1)]12

The above equation indicates the energy productivity change between τ and τ + 1, which can be divided into the following two parts.

(4) MEPIiτ,τ+1=DEτ(Kiτ,Liτ,Eiτ,Yiτ)DEτ+1(Kiτ+1,Liτ+1,Eiτ+1,Yiτ+1)×[DEτ+1(Kiτ+1,Liτ+1,Eiτ+1,Yiτ+1)×DEτ+1(Kiτ,Liτ,Eiτ,Yiτ)DEτ(Kiτ+1,Liτ+1,Eiτ+1,Yiτ+1)×DEτ(Kiτ,Liτ,Eiτ,Yiτ)]12≡EECiτ,τ+1×TCiτ,τ+1

The efficiency index is a dynamic energy efficiency that considers energy use efficiency and technology changes; $EEC_{i}^{\tau ,\tau +1}$ is the change in energy use efficiency and $TC_{i}^{\tau ,\tau +1}$ measures the change in energy technology [64].

Following the study of [65,66], the energy service is expressed as follows:

(5) ES=E×MEPI

### 3.2. Estimating the Rebound Effect

The measurement of the rebound effect is vital for this paper, as it is the basis for determining whether and to what extent a rebound effect occurs in the industry. Critical to iron and steel production are energy services. Therefore, we take energy services, capital, and labor as inputs to the production process and construct the translog cost function in the second stage [20]. The translog cost function has fewer constraints and more alterable substitution elasticity parameters than other functional forms. Moreover, assuming that the producer is rational and the social production can achieve equilibrium, the function contains both factor demand and factor price changes. To mitigate the impact of heteroscedasticity, this study takes logarithms of input and output variables. The cost function is expressed as follows:

(6) $$\ln TC=\alpha_{0}+\kappa_{1}\left(\ln Y\right)+\frac{1}{2}\kappa_{2}{\left(\ln Y\right)}^{2}+\sum_{m}\delta_{m}\ln Y\ln p_{m}+\sum_{m}\alpha_{m}\ln p_{m}+\frac{1}{2}\sum_{m}\sum_{n}\gamma_{mn}\ln p_{m}\ln p_{n}\;\forall m,n=K,L,\mathrm{ES}$$

where TC is the total cost and Y is the industrial output. $p_{m}$ and $p_{n}$ are input factor prices.

Using Shephard’s Lemma, this study finds the partial derivative of the logarithm of the factor prices. The cost share equation is described as:

(7) $$S_{m}^{*}=\frac{\partial \ln TC}{\partial \ln p_{m}}=\alpha_{m}+\sum_{n}\gamma_{mn}\ln(p_{n})+\delta_{m}\ln(Y)\;\forall m,n=K,L,\mathrm{ES}$$

The observed cost shares in practice are affected by the perturbation term. Therefore, the observed cost share equations can be constructed as:

(8) $$\left\{ \begin{array}{l} S_{K}=\alpha_{K}+\gamma_{KK}\ln p_{K}+\gamma_{KL}\ln p_{L}+\gamma_{KES}\ln p_{ES}+\delta_{K}\ln Y+\upsilon_{K}\\ S_{L}=\alpha_{L}+\gamma_{LK}\ln p_{K}+\gamma_{LL}\ln p_{L}+\gamma_{LES}\ln p_{ES}+\delta_{L}\ln Y+\upsilon_{L}\\ S_{ES}=\alpha_{ES}+\gamma_{ESK}\ln p_{K}+\gamma_{ESL}\ln p_{L}+\gamma_{ESES}\ln p_{ES}+\delta_{ES}\ln Y+\upsilon_{ES} \end{array}\right.$$

Substituting $P_{ES}=P_{E}/\zeta$ into the above equation, where ζ represents energy efficiency. The above equations can be rewritten as:

(9) $$\left\{ \begin{array}{l} S_{K}=\alpha_{K}+\gamma_{KK}\ln p_{K}+\gamma_{KL}\ln p_{L}+\gamma_{KES}(\ln p_{E}-\ln\zeta )+\delta_{K}\ln Y+\upsilon_{K}\\ S_{L}=\alpha_{L}+\gamma_{LK}\ln p_{K}+\gamma_{LL}\ln p_{L}+\gamma_{LES}(\ln p_{E}-\ln\zeta )+\delta_{L}\ln Y+\upsilon_{L}\\ S_{ES}=\alpha_{ES}+\gamma_{ESK}\ln p_{K}+\gamma_{ESL}\ln p_{L}+\gamma_{ESES}(\ln p_{E}-\ln\zeta )+\delta_{ES}\ln Y+\upsilon_{ES} \end{array}\right.$$

According to [67], the parameters in the above equations must satisfy symmetry, homogeneity, and summation properties.

(10) $$\begin{array}{l} \gamma_{mn}=\gamma_{nm}\\ \sum_{m}\gamma_{mm}=\sum_{n}\gamma_{nm}=0\;\forall m,n=K,L,ES\\ \sum_{i}\alpha_{m}=1,\;\sum_{m}\delta_{m}=0 \end{array}$$

In this study, the equations’ parameters are estimated using the seemingly uncorrelated regression method [68]. This method considers that there may be correlations between the perturbation terms of the equation and the existence of parameter constraints across equations. According to Equations (9) and (10), this study can obtain the substitution elasticity among production factors by substituting actual observed values. However, these equations may lead to singularities in the perturbation terms. To prevent the homogeneous problem of the interference term, this study removes the capital equation and estimates the remaining equations. Considering that the response of production inputs to price or efficiency changes may be a slow process, a one-period lag term is added to Equation (9).

After estimating the parameters in Equation (9), we focus on the response of producers to changes in input prices. The Allen–Uzawa elasticity of substitution (AES) and cross-price elasticity (CPE) are standard measures of response to price changes [20]. The AES is given as follows:

(11) $$\sigma_{\mathrm{mn}}=\frac{\hat{\gamma_{mn}}+S_{m}\cdot S_{n}-S_{m}\omega_{mn}}{S_{m}\cdot S_{n}}\;\forall m,n=K,L,\mathrm{ES}$$

If m=n, then $\omega_{mn}=1$, otherwise $\omega_{mn}=0$. Then, the own-price elasticity ($\eta_{mm}$) and the cross-price elasticity ($\eta_{mn}$) are given as:

(12) $$\eta_{mm}=\sigma_{mm}S_{m}\;\mathrm{and}\;\eta_{mn}=\sigma_{mn}S_{m}\;\forall m\neq n$$

Based on the study of [65], the direct rebound effect by factor substitution ($RE^{Substitution}$) can be calculated as:

(13) $$RE^{Substitution}=\frac{\partial E}{\zeta }\frac{\zeta }{E}+1=-\eta_{ES,ES}$$

The direct rebound effect through the output channel ($RE^{Output}$) can be estimated by Equation (14).

(14) $$RE^{Output}=\frac{\partial \ln E}{\partial \ln Y}\times \frac{\partial \ln Y}{\partial \ln p_{Y}}\times \frac{\partial \ln p_{Y}}{\partial \ln\zeta }=\frac{\partial \ln E}{\partial \ln Y}\times \frac{\partial \ln Y}{\partial \ln\zeta }$$

The energy rebound effect (RE) can be composed of two parts: one is the factor substitution effect, and the other is the output effect [20]. Therefore, it can be expressed as follows:

(15) $$RE=RE^{Substitution}+RE^{Output}$$

### 3.3. Variables and Data Source

This study selects provincial data to calculate energy efficiency and the energy rebound effect of China’s ISI. Considering data availability and data statistics consistency, we chose 2000–2019 as the sample period (Supplementary Materials). Due to data availability, the study covers 29 Chinese provinces, excluding Hainan, Tibet, Hong Kong, Macau, and Taiwan [69]. These data are from the Statistical Yearbook of China and each province, China Industry Statistical Yearbook, Price Statistical Yearbook, Labor Statistical Yearbook, and CEIC database. To eliminate price change factors and ensure the comparability of economic data over different periods, all economic nominal variables are converted to the year 2000 price level [70,71,72]. Table 1 shows the descriptive statistics of production factors and their prices in the ISI. As can be seen from Table 1, the average industrial output of 29 provinces is about 125 billion Yuan and the standard deviation of industrial output is about 187 billion Yuan. It reflects the unbalanced development of the ISI in different regions of China. The data distribution of each variable is relatively scattered. The iron and steel production factors and prices vary widely in different periods.

The variables used to explore the industrial rebound effect are described as follows.

(1) Industrial output (Y) and its price (PY). Measures of industrial output include the industrial products, industrial value added, and gross output. Due to the variety of products, the gross output value data are taken as the desired output. The data between 2000 and 2016 is from the China Industry Statistical Yearbook, while the data between 2017 and 2019 is from the Statistical Yearbook of each province. It is because after 2016, the China Industry Statistical Yearbook no longer covers gross output data. This study deflates the raw data using the ex-factory industrial price index and converts it to the industrial output value at the constant price in 2000. Its price is expressed by the producer price indices for industrial products. The data are obtained from the China Statistical Yearbook.

(2) Labor (L) and its price (PL). Labor input is measured by the average employment population in the ISI. The labor data comes from the China Industry Statistical Yearbook. The labor statistics of individual years are missing, so we use the linear difference method to fill in. This study uses the average wage of employees as the industry’s labor price, and then deflates it by using the consumer price index to obtain the real labor price in constant with the year 2000 prices. The wage data are from the Labor Statistical Yearbook and the consumer price index is from the China Statistical Yearbook.

(3) Capital (K) and its price (PK). Capital stock is used to measure the capital input. No official statistics on capital stock are published in China. The common method currently used in the academic community is the perpetual inventory method proposed by [73]. We employ this method to estimate the capital stock, which can be expressed as:

(16) $$K_{t}=K_{t-1}\left(1-\delta_{t}\right)+I_{t}$$

where $K_{t}$ and $K_{t-1}$ represent the capital stock in the year t and year t − 1, respectively. $I_{t}$ is the investment in the year t, and $\delta_{t}$ denotes the depreciation rate in the year t. The calculation of capital stock requires four items: the capital stock in the base year, annual investment (data are from the China Industry Statistical Yearbook), fixed asset investment price index (data are from the China Statistical Yearbook), and the depreciation rate. The calculation process is referred to in [74,75]. The capital price is the actual return on fixed assets investment. Capital price can be expressed as the nominal interest rate plus the depreciation rate, and then minus the inflation rate. This study estimates capital price by referring to the method of [21], and the data can be obtained from the CEIC database.

(4) Energy use (E) and its price (PE). The energy consumption of the industry can be used as an energy input. Based on the energy conversion factors, we convert different kinds of energy into standard coal, and then aggregate them to get the total energy use of the ISI. Data for each energy use are obtained from the Statistical Yearbook of each province and data for conversion factors comes from the China Energy Statistical Yearbook. For some provinces without energy data reports, we draw on the studies of [76,77] for estimation. The available official data generally contain statistics on energy consumption and do not include statistics on energy prices. At present, China’s energy price system is not yet sound. Electricity and natural gas prices are mainly set by the government, and coal prices are integrated with the market. Therefore, this study adopts each province’s fuel and power purchase price index to measure energy prices, which comes from the Statistical Yearbook of each province.

## 4. Empirical Discussion

### 4.1. The Result of Dynamic Energy Efficiency

Table 2 reports the dynamic energy efficiency and its decomposition indicators. MEPI is the dynamic energy productivity of the ISI. Energy efficiency change (EEC) measures the catch-up effect. It shows the change in the relative energy performance of a provinces’ production frontier from one period to another. Technology change (TC) is the frontier-shift effect. It measures the movement of a provinces’ production technology from one period to another [63,64]. At the national level, the average industrial energy efficiency between 2000 and 2019 is 1.080. This indicates that energy productivity keeps improving during the sample period. It also indirectly reflects the achievement of the energy conservation policies of the ISI in recent years. Technology change (TC) is essential in promoting energy efficiency, which shows an obvious frontier-shift effect. While the contribution of the energy utilization efficiency (EEC) is 0.998, which shows that the catch-up effect of the ISI needs to be strengthened. In addition to enhancing R&D innovation and technology introduction, the ISI should also improve its management and production structure. Firms need to avoid sloppy production and reduce the energy waste caused by human factors [76].

In terms of regions, the eastern region shows the best efficiency performance at 1.106, higher than the national average. It is driven by an improved energy use efficiency and technical progress in the eastern area. This region has the advantage of an obvious catch-up effect and frontier-shift in steel production. This finding is in line with reality [78]. With a high economic development degree in the east, iron and steel firms have more funds to invest in the research and introduction of new technologies. This allows firms to update production equipment and drive energy technology advances. In addition, the eastern region has a high degree of marketization and firms adopt modern management to carry out intensive and refined management, effectively reducing the discharge of energy waste. The energy efficiency performance of the industry in the central region is inferior, mainly due to the progress of energy technology in recent years. Although the energy efficiency in the western region has been improving in recent years, it lags behind that of eastern and central China. According to index decomposition, it can be found that the main reason is the low catch-up effect in western China. The ISI should adopt more mechanized modern equipment and strengthen the management of energy use. In summary, the energy efficiency of the ISI can be improved through high and new technology and scientific management combined with sound policies.

The Five-Year Plan is an important part of China’s national economic plan. The goals put forward by it have a great influence on economic departments. Comparing energy efficiency differences in each period can provide further insight into the energy management of the ISI. Referring to the method of [60], we calculate the accumulated energy efficiency in different periods (Table 3). From the 10th to 13th Five-Year Plans, the accumulated energy efficiency of the ISI shows an upward trend. Previous Five-Year Plans emphasize the green development of industrial sectors, which has a guiding role in the energy conservation of the ISI. In different Five-Year Plans, technology change drives energy efficiency improvement. The country attaches great importance to innovation, and it is expected that technological progress will contribute more to the high-quality development of the ISI in the future. Except for the 12th Five-Year Plan, the accumulated EEC in other periods is less than one. It reflects that the catch-up effect of the ISI is relatively weak. Therefore, energy efficiency improvement requires more emphasis on energy utilization efficiency.

### 4.2. The Estimation Results of the Rebound Effect

After obtaining the energy efficiency, we can calculate the effective energy service and substitute it into the production function as an input factor. This study uses seemingly uncorrelated regression to the equations for the labor and effective energy service. The estimated results of the parameters are reported in Table 4. We do not directly estimate the capital cost share equation to avoid singularity. According to the constraints of symmetry, homogeneity, and the summation properties of the parameters, the parameters in the capital share equation can be calculated accordingly. Two important messages can be obtained from Table 4. First, the lagged one-period variables of labor and energy service shares are significantly positive in the regression estimates. This implies that there is a slow adjustment process for these factor inputs. The labor and energy service in the ISI is somewhat locked in. Thus, it can be seen that the dynamic model, considering the lagged period, is more suitable for this study than the static model [68]. Moreover, the parameters of the share equations for the labor and energy service are significant at the 1% level and their R-square estimates are 0.628 and 0.800, respectively. This demonstrates both the solid explanatory power of the model results and provides convincing evidence for estimating the price elasticity and rebound effects in the next step.

We further calculate the price elasticity of each input factor, which is presented in Table 5. The own-price elasticity of capital, labor, and energy services is −0.1970, −0.7491, and −0.3700, respectively. It indicates that with the price of capital, labor, and energy services rising, the ISI reduces the use of these inputs. The finding is consistent with the microeconomic theory. In terms of the scale of price elasticity, capital has the smallest absolute value. The price increases of capital have relatively little impact on the demand for capital factors. Large-scale changes in the capital in the ISI are not easy to occur in the short run. Due to the long equipment lifetime, firms usually find it difficult to drastically adjust heavy industry production in a short period [79]. The labor demand is more sensitive to its price changes. It indicates that the demand for labor in the industry is more elastic in relation to capital and energy services. In addition, the cross-price elasticity among input factors is positive, and there is substitutability between these factors. Iron and steel firms can use more of an input to replace other inputs. Lower prices for energy services will dampen the demand for capital and labor. The price elasticity of input factors provides the basis for the rebound effect estimation.

**Figure 3:**
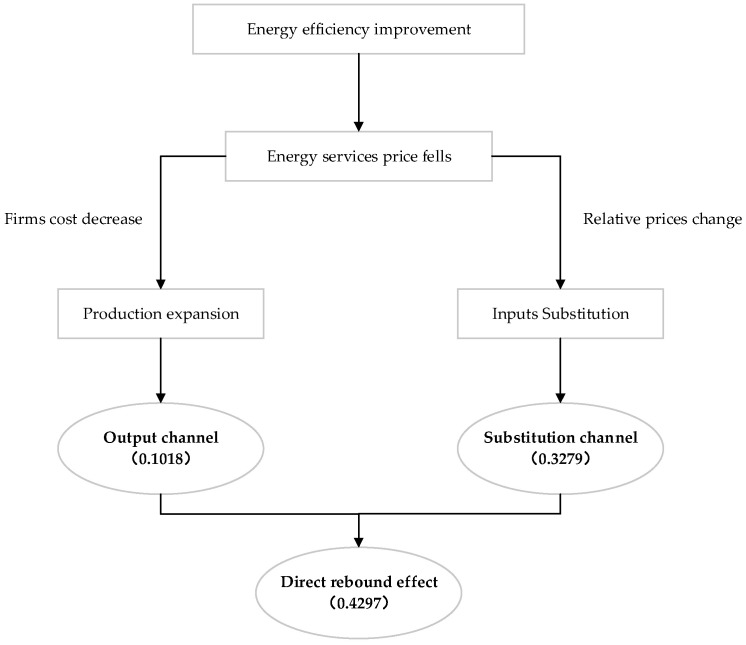
The influence mechanism of the industrial energy rebound effect.

The energy rebound effect is caused by energy services allocation due to changes in the relative price under technical progress. To understand the mechanism of the rebound effect more intuitively, Figure 3 presents the influencing mechanism framework. The rebound effect is the joint influence of the substitution and output channels [80]. It is caused by the process of minimizing cost and maximizing utility when energy efficiency is improved. Energy efficiency improvements in the ISI reduce the energy services price. In this case, firms respond to energy services price changes in two ways. First, because of the substitution elasticity between different input factors, iron and steel firms use relatively cheap energy services to replace other inputs. They increase the energy services input and reduce the investment in non-energy factors. This generates the substitution effect, which reduces the production costs of firms. Second, the decline in energy services prices also makes it cheaper for firms to produce, expanding the boundaries of firms’ production. The production expansion further stimulates the demand for energy services, causing the output effect. In summary, the firms provoked the energy consumption rebound through factor substitution and output expansion.

The industry’s average nationwide rebound effect is 0.4297, showing a partial rebound effect and verifying the H1. The potential energy conservation is realized at 57.03%, which indicates a large space for energy savings. The reason for this is that energy efficiency leads to a reduction in energy services prices. Under the imperfect energy price system, it is inevitably difficult to curb energy consumption to meet the increasing production demand. The additional energy services input is stimulated under the effect of factor substitution and output expansion. The rebound effect from the substitution channel is 0.3279, and from the output channel is 0.1018. It is clear that the substitution channel is more significant than that output channel (H2 is verified). The rebound effect of the ISI is mainly driven by factor substitution, while output expansion plays a relatively minor role. Lower energy services prices incentivize iron and steel firms to engage in factor substitution [71]. The small output effect may be because it is not easy to expand production in the short term. Iron and steel firms are limited in expanding production by capital and labor. China’s industrial policies and energy conservation measures also constrain energy services use. Under the background of steel capacity reduction, there is little room for sustained output growth. Environmental regulation and energy conservation measures make it difficult to realize the output effect [81]. It is necessary to compare the results of this study with the existing research. The rebound effect of the ISI is slightly higher than that of the metallurgical industry [68] and heavy industry in China [20]. In the production process of iron and steel, energy is used as raw material and fuel. The ISI is more dependent on energy and price changes in energy services that have a more significant impact on production.

China is a vast country with significant economic differences across its regions. Analyzing the rebound effect from the whole country may overlook the heterogeneity of regional development. Therefore, we further explore the rebound effect of the ISI in each province. Table 6 shows that the rebound effect in central China is the strongest, slightly higher than the national average (H3 is verified). The central region has 44.93% of the potential energy savings offset by energy efficiency improvements. The higher rebound effect in the central area is inextricably linked to the regional advantages of energy resource endowment. The central region has abundant energy resources such as coal, which greatly enhance the ease of energy investment for iron and steel firms. The central region is also geographically convenient to receive industrial shifts from the eastern region. The industrial expansion in this region leads to a higher dependence on energy service factors. The rebound effect in the eastern and western areas is 42.87% and 41.29%, respectively. Although the rebound effect is low in both areas, the reasons may differ. Due to the higher economic development and better energy market-oriented system in the eastern region, the market can reflect the energy services price. As a result, the effect of changes in the relative price of energy services on other inputs substitution is insignificant and does not cause a surge in energy consumption in the eastern region. Moreover, environmental regulations are strict in the east. The iron and steel firms are unlikely to expand the scale of their output. The lowest rebound effect is observed in the western region. This may be due to the relatively backward economy in this region. From the demand perspective, the product market in the west region is small, constrained by insufficient consumer demand. The small production scale of firms does not stimulate a sharp output expansion even if the energy services price is reduced. The comparison of different regions shows that the imbalance of regional economic development and industrial policy affects the role of energy efficiency on energy conservation [47].

Table 6 also shows that the rebound effect of the ISI in each province is less than 100%, and there is no backfire effect. On the one hand, this affirms that the industry does gain from technological progress. Energy efficiency improvement plays a certain role in energy conservation. On the other hand, it indicates that achieving energy conservation through energy efficiency has not fully committed the expected effect in the ISI. Energy efficiency is an essential way of energy conservation, but it cannot be regarded as the only means for the industry. Instead, other supporting reforms are needed to reduce energy use and protect the environment. The rebound effect should be considered in the industrial energy policy design to ensure that the maximum policy effectiveness can be realized. The research results of the ISI can serve as an important basis for policy making.

China’s ISI is the industrial sector with a very high energy consumption and pollutant emission. With environmental deterioration and energy shortage constraints, this industry is facing a severe situation. How to achieve high-quality development while reducing energy consumption has become a major problem to be solved urgently by the government and industry. At present, the energy conservation policy of the ISI is mainly implemented by administrative controls. Although this approach may produce energy-saving effects in the short term, the economic and social costs are relatively high. In this case, firms lack incentives for long-term energy efficiency improvement. The existence of the rebound effect highlights the importance of a market-oriented approach [82]. The theory of the energy rebound effect provides a new way to study the energy and environmental protection of the ISI under the background of efficiency improvement. While we recognize the government’s regulatory policies, we cannot ignore that the market plays a fundamental role in resource allocation.

## 5. Conclusions and Suggestions

### 5.1. Conclusions

Energy efficiency is an essential means of energy saving and environmental protection. However, the energy efficiency will bring about a rebound effect. This article explores the energy rebound effect of the ISI with the improved method proposed by [20]. The study is conducted in two steps. We construct the DEA model to measure energy efficiency in the first step; in the second step, we calculate the industrial energy rebound effect between 2000 and 2019. The research draws the following conclusions.

(1) The dynamic energy efficiency of the ISI is 1.08. Its improvement is driven by energy technology progress. The best energy efficiency performance in eastern China is driven by the combination of energy use efficiency improvement and energy technology progress. The central region has the second-best energy efficiency performance while the western region has the lowest energy use efficiency, resulting in their energy efficiency performance lagging behind other areas. The green transformation of the ISI needs to pay attention to improving energy efficiency. It requires great effort in energy utilization and energy technology. On the one hand, efficient energy utilization can be realized by adopting modern production management. On the other hand, firms can strengthen the invention and promotion of new metallurgical technology in production.

(2) The energy rebound effect does exist in iron and steel production. It means that energy efficiency improvements do not bring about the expected energy use reduction. The average rebound effect is 0.4297, showing a partial rebound effect. Potential energy savings from improved energy efficiency reached 57.03%. Although energy efficiency improvements lead to the rebound effect, the energy backfire does not occur. It indicates that in most cases, the amount of energy saved by efficiency improvement is greater than the amount of energy rebound. Thus, improving energy efficiency remains an effective way to save industrial energy use. As for the decomposition, factor substitution is more critical in promoting the rebound effect, with a value of 0.3279, and the impact of an output increase on the rebound effect is 0.1018. This finding highlights the role of the rebound effect in the green economy of the ISI. Ignoring the rebound effect will greatly diminish the effectiveness of green transition policies and actions. A market-oriented reform of production factors, especially energy prices, will help promote high-quality development of the ISI.

(3) In terms of provincial distribution, the rebound effect shows regional heterogeneity. It is relatively concentrated in the central provinces. The reason is that the central provinces have better resource endowments and the convenience of accepting industrial transfer. The rebound effect of the western region is the lowest, which is related to the small scale of steel production and the lagging technology in this region. Economic development, resource endowment, and industrial policy are the critical factors leading to regional differences. This reveals that the high-quality and green development of the ISI needs to optimize the regional layout. Market demand, resource guarantee, and environmental capacity are key factors for firms to consider in the future.

### 5.2. Policy Suggestions

This paper explores the energy efficiency and rebound effect of China’s ISI. According to the conclusion, we put forward suggestions to address the energy conservation and environmental protection challenges.

First, it is urgent to enhance industrial energy efficiency through technological advances. The energy savings are the result of the combined effect of the theoretical energy saving and the rebound amount brought by energy efficiency. Technical innovation is responsible for current production efficiency and energy consumption. Therefore, iron and steel firms need to improve their independent innovation capability in the low-carbon economy. Firms should strengthen investment in energy-saving production technology research and development. Firms can further update advanced technology equipment and eliminate high energy-consuming obsolete equipment to improve energy efficiency. In addition, firms should pay attention to management innovation, system optimization, and other soft technological advances for effective resource allocation. This is because it can better raise awareness of energy conservation and promote energy efficiency improvements by managing the optimal use of energy elements. For the country, the government should watch out for the technology spillover effect between regions and technology transfer [83,84]. The government can guide iron and steel firms to bring advanced technologies from the east to the other areas and promote energy efficiency in the west region.

Second, the government should improve the policies’ effectiveness and focus on energy prices in the ISI. This study finds a high rebound effect in the ISI. Governments and firms need to become aware of the rebound effect gradually. The rebound effect affects how energy efficiency improvements reduce energy consumption [85]. It also closely relates to the effectiveness of energy policies [21]. If the ISI focuses on enhancing energy efficiency and does not involve energy price reform, the policy effect will be less than expected. The development and evaluation of policies in China often ignore the elasticity of energy services by industry. In this case, the effectiveness of the policy is reduced. The decrease in the price of energy services is responsible for the energy consumption rebound, so the rebound effect can be reduced by adjusting the energy services price. The primary concern is to rationalize energy prices so that they can reflect the cost and scarcity of energy. The current low energy price policy is contrary to the goal of energy conservation [82]. Therefore, it is necessary to accelerate the energy price and taxation reform. From the market means, the government needs to adjust energy prices moderately to mitigate the energy rebound effect. From the administrative means, the government should expand the energy tax policy regulation space. Through taxation, energy consumption can be regulated to suppress the rebound effect [86].

Third, the ISI should consider regional differences in developing energy efficiency policies. The rebound effect in the ISI is regionally heterogeneous, and improving energy efficiency generates different levels of demand for energy services. Differentiated energy conservation policies should be formulated in the context of regional development. High energy-consuming equipment should be restricted in the central region with a rich resource endowment. Meanwhile, the central area should change the energy structure dominated by coal consumption and strengthen clean energy utilization. The additional energy consumption will be added, provided the rebound effect exists. The problems of resource depletion and environmental pollution are expected to be ameliorated if the energy mix is rationalized so that non-fossil energy sources are the main component of the increased energy consumption. Iron and steel firms can establish a clean production system and introduce more renewable energy generation through energy storage [87]. As the receiving region of industrial transfer, the western region should learn from the development experience of the eastern region and improve energy efficiency while focusing on the energy rebound effect.

This research provides a useful reference for future research on the energy rebound effect of China’s industrial sectors. At the end of the article, we need to point out that there may be some flaws and limitations in the study. First, coal energy and capital prices are regulated by the Chinese government and not fully determined by the market. Limited by data availability, this study cannot obtain data on the energy and capital prices of the ISI. We apply the provincial fuel and power purchase price index and capital price as the proxy. These data may affect the result of energy efficiency and the rebound effect. Second, the estimation of energy efficiency is the basis for the rebound effect. There are other alternative methods, such as the stochastic frontier approach, that can estimate energy efficiency. Moreover, the selection of different production functions and input factors may also affect the rebound effect estimation. These questions need to be considered in future research. With the deepening of market-oriented reform, more comprehensive data will provide new support for the research. Additionally, with new methods, the industrial rebound effect estimation will be more accurate and closer to the actual situation. In future studies, calculating the rebound effect of different energy types in the ISI may yield more findings.

## Figures and Tables

**Table 1:** Descriptive statistics of variables.

Variable (Abbreviation)	Unit	Size	Mean	Std. Dev.	Min	Max
Industrial output (Y)	Hundred million Yuan	29	1245.151	1867.287	10.750	13,960.170
Labor (L)	Ten thousand persons	29	12.256	12.558	0.860	77.780
Capital (K)	Hundred million Yuan	29	542.098	714.011	5.359	5085.804
Energy (E)	Million Tce	29	17.638	25.311	0.380	202.391
Industrial price (PY)	Price index	29	127.747	29.677	84.323	254.786
Labor price (PL)	Thousand Yuan	29	27.135	13.095	5.715	81.156
Capital price (PK)	Price index	29	9.967	2.170	0.150	15.657
Energy price (PE)	Price index	29	180.635	60.276	96.018	441.993

**Table 2:** Dynamic energy efficiency and its decompositions of ISI (2000–2019).

Province	MEPI	EEC	TC
Beijing (E)	1.125	1.028	1.089
Fujian (E)	1.093	0.989	1.107
Guangdong (E)	1.110	0.990	1.123
Hebei (E)	1.077	0.997	1.089
Jiangsu (E)	1.135	1.014	1.122
Liaoning (E)	1.077	1.003	1.079
Shandong (E)	1.119	1.016	1.114
Shanghai (E)	1.095	0.993	1.113
Tianjin (E)	1.130	1.006	1.120
Zhejiang (E)	1.099	1.002	1.097
Anhui (C)	1.128	1.049	1.082
Heilongjiang (C)	1.070	1.010	1.061
Henan (C)	1.124	1.040	1.089
Hubei (C)	1.058	0.986	1.083
Hunan (C)	1.061	0.991	1.074
Inner Mongolia (C)	1.057	0.993	1.072
Jiangxi (C)	1.051	0.978	1.082
Jilin (C)	1.028	0.958	1.077
Shanxi (C)	1.059	0.994	1.071
Chongqing (W)	1.091	1.025	1.066
Gansu (W)	1.042	0.963	1.083
Guangxi (W)	1.120	1.038	1.084
Guizhou (W)	1.066	1.002	1.072
Ningxia (W)	1.036	0.972	1.069
Qinghai (W)	0.948	0.895	1.070
Shaanxi (W)	1.188	1.077	1.140
Sichuan (W)	1.049	0.986	1.072
Xinjiang (W)	1.058	0.981	1.078
Yunnan (W)	1.030	0.971	1.068
East China mean	1.106	1.004	1.105
Central China mean	1.071	1.000	1.077
West China mean	1.063	0.991	1.080
China mean	1.080	0.998	1.088

Note: MEPI, EEC, and TC represent dynamic energy efficiency, energy utilization efficiency change, and energy technology change, respectively.

**Table 3:** Accumulated energy efficiency and its determinants during each Five-Year Plan.

Period	Year	MEPI	EEC	TC
10th Five-Year Plan	2001–2005	1.469	0.844	1.734
11th Five-Year Plan	2006–2010	2.371	0.825	2.820
12th Five-Year Plan	2011–2015	3.660	1.031	3.470
13th Five-Year Plan	2016–2019	4.029	0.876	4.455

**Table 4:** The results of the cost share equations.

Variables	SL	SES
lagSL	0.807 *** (0.000)	
lnPK	−0.007 *** (0.000)	−0.094 *** (0.000)
lnPL	0.012 *** (0.000)	−0.005 *** (0.000)
lnPES	−0.005 *** (0.000)	0.099 *** (0.000)
lnY	−0.002 *** (0.000)	−0.014 *** (0.000)
lagSES		0.742 *** (0.000)
Constant	0.025 *** (0.000)	−0.082 *** (0.000)
Observations	521	521
R^2^	0.628	0.800

Note: *** *p* < 0.01. t values are shown in parentheses.

**Table 5:** Price elasticity of input factors in ISI.

*η_ij_*	K	L	ES
K	**−0.1970**	0.5400	0.3249
L	0.0520	**−0.7491**	0.0451
ES	0.1451	0.2091	**−0.3700**

Note: The bold type represents own-price elasticity.

**Table 6:** The average energy rebound effect from 2000 to 2019.

Eastern Province	Eastern RE	Central Province	Central RE	Western Province	Western RE
Beijing	0.3057	Anhui	0.4731	Chongqing	0.4330
Fujian	0.4550	Heilongjiang	0.4319	Gansu	0.4585
Guangdong	0.4072	Henan	0.4304	Guangxi	0.4220
Hebei	0.4119	Hubei	0.4602	Guizhou	0.3409
Jiangsu	0.4741	Hunan	0.4605	Ningxia	0.3075
Liaoning	0.4739	Inner Mongolia	0.4730	Qinghai	0.4408
Shandong	0.4739	Jiangxi	0.4279	Shaanxi	0.4520
Shanghai	0.3468	Jilin	0.4639	Sichuan	0.4738
Tianjin	0.4684	Shanxi	0.4231	Xinjiang	0.4111
Zhejiang	0.4704			Yunnan	0.3895
East mean	0.4287	Central mean	0.4493	West mean	0.4129

## Data Availability

The data used in this study are available in the Statistical Yearbook of China and each province, Price Statistical Yearbook, Labor Statis-tical Yearbook, and CEIC database. These data can be found at the website: https://data.cnki.net/Yearbook; https://insights.ceicdata.com/ (accessed on 12 May 2022).

## Supplementary Materials

The following supporting information can be downloaded at: https://www.mdpi.com/article/10.3390/ijerph191811767/s1.
